# Research and Publication Ethics in Journal of Functional Morphology and Kinesiology

**DOI:** 10.3390/jfmk5020042

**Published:** 2020-06-10

**Authors:** Grazia Maugeri, Giuseppe Musumeci

**Affiliations:** 1 Department of Biomedical and Biotechnological Sciences, Human, Histology and Movement Science Section, University of Catania, Via S. Sofia n°87, 95123 Catania, Italy; graziamaugeri@unict.it; 2 Research Center on Motor Activities (CRAM), University of Catania, Via S. Sofia n°97, 95123 Catania, Italy; 3 Department of Biology, Sbarro Institute for Cancer Research and Molecular Medicine, College of Science and Technology, Temple University, Philadelphia, PA 19122, USA

**Keywords:** statistical analysis, experimental approach, design, ethical standard, best practice, sample size, performance indicators, reliability of the measures, conflict of interest, statement, author contributions, plagiarism, funding

## Abstract

Research is required to minimize uncertainty and to be reproducible, that is, the design, implementation, evaluation, interpretation, and reporting of the presented data, must follow a good practice. An appropriate experimental design, an accurate execution of the study, a strict criticism of the obtained data while avoiding overestimation, as well as a suitable interpretation of main outcomes, represent key aspects in reporting and disseminating research to the scientific community. Furthermore, author contribution, responsibility, funding, acknowledgement, and adequately declaring any conflict of interest play important roles in science. The *Journal of Functional Morphology and Kinesiology* (*JFMK*), a member of the Committee on Publication Ethics (COPE), is committed to the highest scientific and ethical standards and encourages all authors to take into account and to comply, as much as possible, with the contents and issues reported in this technical note. This could be useful to improve the quality of the manuscripts and avoid misconduct, as well as to stimulate interest and debate, reflecting upon uses and misuses within our disciplines belonging to the medicine area (sports medicine and movement sciences) categories: anatomy, histology, orthopedics and sports medicine, rheumatology, sports sciences, physical therapy, sports therapy, and rehabilitation.

## 1. Introduction

Over the years, the scientific world, as well as associated ethical issues, have been rapidly evolving. In this document, we report the best research practices, taking into consideration ethical aspects and international laws, in order to promote high-quality research and its integrity. The reported guidelines in the present document ensure that submitted research within the vast, interdisciplinary field of the medicine (sports medicine and movement sciences), categories: anatomy, histology, orthopedics and sports medicine, rheumatology, sports sciences, physical therapy, sports therapy, and rehabilitation, meet community-agreed standards in term of quality and integrity. *JFMK* is a member of the Committee on Publication Ethics (COPE) [1]. *JFMK* adopts a zero-tolerance policy in the case of results falsification and inappropriate authorship credit. Furthermore, to guarantee high-quality scientific studies and avoid plagiarism, *JFMK* adopts a strict peer review process and it makes use of iThenticate [2] to check submissions against previous publications. Plagiarism comprises copying text, ideas, images, or data from another document, including your own previous publications. The reuse of text that is copied from another source must be between quotes and the original document must be cited in the text. It is mandatory to cite in the document if the experimental design or the structure or language has been inspired by previous works. The detection of plagiarism during the peer review process can cause the rejection of work. If plagiarism is detected after publication, *JFMK* may publish a correction or retract the paper.

Authors wishing to publish their documents in *JFMK* must abide by the following:

- Any facts that may represent a possible conflict of interest of the author(s) must be disclosed in the document prior to submission;
- Authors should critically show their research findings and propose an objective discussion of the significance of their results;
- To ensure the reproducibility of results, data and methods performed in the research must be presented in detail;
- Place your power analyses in the manuscript to obtain an "n sample" adapted to the study;

Ensure that statistical analysis using the reliability of the dependent measures with intra-class correlation coefficients and ∆% (from two trials). *JFMK* strongly encourage the use of ANOVA and *t*-tests; Bland and Altman tests are recommended for assessing the agreement between two methods of clinical measurement; Pearson, Kendall, and Spearman for correlation studies; factorial analysis when subjects are “> 100”;

- The results must be clear, intuitive, and not duplicated in table/s and vice versa;
- Raw data have to be available for presentation to referees and/or editors of the journal if requested. Authors need to preserve raw data for a reasonable time after publication;
- A concise and explanatory cover letter must be included with each manuscript submission. It should describe the aim and content of the document according to the scope of the journal.

The submission of a manuscript to *JFMK* implies that the study described has not been published before and that it is not under consideration for publication anywhere else. Its content, figures, and tables have not been published or submitted elsewhere in print or electronic format and will not be submitted elsewhere during the period of review. The republishing of non-novel content (e.g., an English translation of a paper that is already published in another language) is not tolerated. The main types of scientific contributions include:

- Original Articles: These provide novel scientific information, whose quality and impact will be considered during the peer review process. Authors are encouraged to not divide their study in different related manuscripts, although Short Communications of preliminary, but significant, results will be taken into consideration.
- Reviews: These provide concise and precise updates on the latest progress made in a given area of research e.g., narrative review. Systematic reviews should follow the Preferred Reporting Items for Systematic Reviews and Meta-Analyses (PRISMA) guidelines [3].
- Observational study: This provides nonexperimental data and it should follow Strengthening the Reporting of Observational Studies in Epidemiology (STROBE) guidelines. See strobe-statement.org for statements and/or explanations.
- Meta-analyses: These should be performed according to PRISMA, Quality of Reporting of Meta-analyses (QUOROM), and Meta-analysis of Observational Studies in Epidemiology (MOOSE) guidelines.
- Case reports: These should comprise an accurate report of the symptoms, signs, diagnosis, treatment, and outcomes of patients. Case reports usually show findings that shed new light on the possible pathogenesis of a disease or an adverse effect, as well as new therapeutic approaches [4].

If authors include already published figures or images, it is necessary to obtain the permission from the copyright holder to publish under the CC-BY license. All submitted image files must conform to the original. Manipulations and/or adjustments are not allowed. The irregular manipulation comprises the grouping of different images that should be shown separately (e.g., merging different parts of distinct gels), and modifying the contrast, brightness, or color balance to obscure, eliminate, or enhance some details. If irregular image manipulation is detected during the peer review process, the manuscript can be rejected. The detection of image manipulation after publication can cause a correction or retraction of the paper. Authors are expected to comply with the best ethical publication practices when publishing in *JFMK*. It is important that any type of investigation adheres to research ethics in order to ensure the credibility of the scientific community and the perception of the readers to judge and accept new findings.

### 1.1. Research Involving Human Subjects

Research involving human subjects, must to be conducted with respect to the participants’ rights, safety, and wellbeing. When reporting on research that involves human subjects, human material, human tissues, or human data, authors must declare that all the investigations were performed in accordance with the Declaration of Helsinki [5]. As reported in point 23 of this declaration, research involving human subjects should to be approved by an ethical committee [6]. In the methods section, authors have to indicate the project identification code, date of approval, and name of the ethics committee or institutional review board. Data relating to individual participants must be described in detail, except private information not relevant to the research and which could identify participants. Research involving human participants also has to include written informed consent from participating patients/individuals in the study who can be identified. The informed consent should be exhaustive and clearly readable, but patient details must be anonymized (e.g., do not mention specific age, ethnicity, or occupation). For manuscripts containing these details, i.e., personal information, and/or images of patients, it is mandatory to obtain signed informed consent from patients before submitting to *JFMK*. For subjects aged < 18 years, the informed consent must be provided with appropriate written documentation by a parent/guardian/tutor. The information regarding the informed consent should be stated in the manuscript and not submitted as separate files, only in case of request should they be provided to the editorial office. It is necessary to provide, if it exists, information regarding the funding/sponsor, or other potential conflicts of interest of the participants (which should be clarified and detailed). For the purposes of publishing in *JFMK*, consent should comprise unlimited permission for publication in all different formats and in sublicensed and reprinted versions.

### 1.2. Research Involving Animals

Research involving animals must be conducted ethically in accordance with the principles of the Code of Practice for the Housing and Care of Animals Used in Scientific Procedures [7]. The acquisition of animals has to follow the extant legal statutes and regulations. The safety and the wellbeing of the animals has to be guaranteed in all phases of research by qualified personnel, to reduce, as much as possible, the discomfort and pain of the animals. Manuscripts containing original research involving animals must indicate details of approval by a properly constituted research ethics committee [8]. Authors should indicate, in the methods section, the project identification code, date of approval, and name of the ethics committee or institutional review board [9]. *JFMK* endorses the Animal Research: Reporting of In Vivo Experiments (ARRIVE) guidelines [10] for experiments using live animals. The editors will require that the potential benefits of research needing animals is scientifically significant. Moreover, the performed research should be in accordance with the “3Rs”, i.e., the substitution of animals wherever possible; the use of the least possible number of animals; experimental procedures to decrease, as much possible, the pain of animals [11].

### 1.3. Research Involving Cell Lines

Authors submitting research with cell lines should indicate, in the methods section, the origin of any cell lines. For established cell lines, the provenance should be indicated, and appropriate references of published papers or commercial sources must also be reported [12]. Researchers working on cell lines should pay attention to cell contamination with microorganisms, genetic and phenotypic cell line instability, cell line development, acquisition, authentication, cryopreservation, and transfer between laboratories, and characterization. Research involving de novo cell lines [13], including those gifted from another laboratory [14], must provide details of an institutional review board or ethics committee approval. Moreover, it is mandatory provide written informed consent if the cell line is of human origin.

### 1.4. Research Involving Plants

Research involving plants (cultivated or wild or in a collection of plant material) must be conducted in accordance with the Convention on Biological Diversity [15] and the Convention on the Trade in Endangered Species of Wild Fauna and Flora [16]. Authors must provide genetic information and origin [17]. For each submitted manuscript involving rare and non-model plants, voucher specimens must be collected in a herbarium or museum, in order to be accessible for future investigators. They should report information on the site of collection by specifying GPS coordinates and date of collection. A waiver of this requirement for locality information may be granted for rare, threatened, or endangered species, but authors must to describe this in the cover letter.

### 1.5. Research Involving the Use of Bio-Banks

Research involving the use of bio-banks must to conducted in accordance with ethical, legal, and social issues (ELSI) [18].

### 1.6. Research Involving Sports Medicine and Movement Sciences

In the specific field of sports medicine and movement sciences, research/surveys and other contributions can be conducted by consulting specific databases (e.g., SPORTDiscus, CINAHL, CSA Physical Education Index) [19,20,21]. 

### 1.7. Authorship

Manuscripts submitted to *JFMK* should following the International Committee of Medical Journal Editors (ICMJE) guidelines [22], according to which the co-authors of a work have to be directly involved in three of the following:

- the planning and contribution of some component of the study (conception, experimental design, conduct, analysis, or findings interpretation) which led to the paper;
- writing a draft of the paper or revising it for intellectual content;
- final approval of the version to be published. All authors should review and approve the article before it is submitted for publication.

Those authors who contributed to the work but do not qualify for authorship should be included in the acknowledgements.

### 1.8. Conflicts of Interest

All authors have to meet the authorship conditions, and any change to the author list should be approved by all of them. The corresponding author should inform and involve all authors in major decisions about the publication. The corresponding author must also include, in a separate section of the document, the summary statement “Conflicts of Interest”, reflecting all the collected potential conflict of interest disclosures in the form. As reported in the ICMJE guidelines, “Authors should avoid entering into agreements with study sponsors, both for-profit and non-profit, that interfere with authors’ access to all of the study’s data or that interfere with their ability to analyze and interpret the data and to prepare and publish manuscripts independently when and where they choose". The authors must disclose all relationships or interests that could unsuitably influence their study. Potential conflicts of interest comprise financial interests, e.g., honoraria, grants, or other funding, and non-financial interests, like personal relationships and affiliations. The authors are encouraged to include the following sentence: the funder/sponsor had no role in the design and conduct of the study; collection, management, analysis, and interpretation of the data; preparation, review, or approval of the manuscript; or the decision to submit the manuscript for publication.

### 1.9. Clinical Trials Registration

Clinical trials can be pre-registered with the international clinical trials register. *JFMK* endorses different databases, such as the EU Clinical Trials Register or others listed by the World Health Organization International Clinical Trials Registry Platform. *JFMK* endorses the Consolidated Standards of Reporting Trials (CONSORT) Statement [23] to ameliorate the reporting of randomized controlled trials (RCT). All documents must comply to the Enhancing the QUAlity and Transparency Of health Research (EQUATOR) Network [24], which promotes the transparent and accurate reporting of health research studies and aims to enhance the value and reliability of medical research literature.

Submission process guidelines. https://www.mdpi.com/editorial_process#standards.

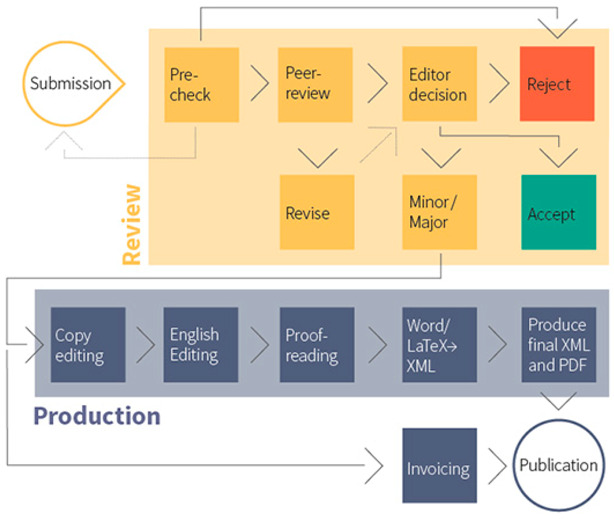



## 2. Summary

In summary, this document aims to help authors understand the integrity and ethical issues of research and publication and to avoid misconduct or distracting mistakes in papers related to sports medicine and movement sciences submitted to *JFMK*. To achieve the maximum effect within the scientific community, all editors and authors should comply to universal standards and good practices. Authors must ensure the integrity of the submitted study and conduct studies in humans, animals, or others ethically and in accordance with *JFMK* guidelines. The scientific community must be based on truth, responsibility, innovation, openness, and transparency. Given the importance and impact of research on society, a high level of ethical awareness is required for authors who aspire to publish academic papers in our journal.
